# Bioinformatics Resources and Tools for Phage Display

**DOI:** 10.3390/molecules16010694

**Published:** 2011-01-18

**Authors:** Jian Huang, Beibei Ru, Ping Dai

**Affiliations:** Key Laboratory for Neuroinformation of Ministry of Education, School of Life Science and Technology, University of Electronic Science and Technology of China, Chengdu, Sichuan 610054, China; Email: rubeibei1988@gmail.com (B.R.); pingdai@263.net (P.D.)

**Keywords:** phage display, mimotope, database, algorithm, software

## Abstract

Databases and computational tools for mimotopes have been an important part of phage display study. Five special databases and eighteen algorithms, programs and web servers and their applications are reviewed in this paper. Although these bioinformatics resources have been widely used to exclude target-unrelated peptides, characterize small molecules-protein interactions and map protein-protein interactions, a lot of problems are still waiting to be solved. With the improvement of these tools, they are expected to serve the phage display community better.

## 1. Introduction

Phages, also known as bacteriophages, are viruses that infect bacterial cells. Many phages such as M13 and fd are good expression vectors. In 1985, George P. Smith displayed foreign peptides on the virion surface by inserting the foreign DNA fragments into the filamentous phage gene III [1]. It was demonstrated that foreign peptides in fusion proteins on the virion surface were in immunologically accessible form and specific fusion phage could be enriched by affinity selection [1]. Also in that paper, Smith, considered the father of phage display technology, inferred that desired clones could be isolated from a phage library of random inserts in a fusion-phage vector by one or more rounds of selection [1].

Since the pioneering work described above, phage display technology has further been developed and improved by scientists from various fields, and its applications has extended from epitope mapping to antibody engineering and organ targeting [2,3,4,5]. So far, phage display technology has been widely used in basic research such as studying the sites and the networks of protein-protein interactions [6,7,8,9], and in applied research such as developing new diagnostics, therapeutics and vaccines [10,11,12,13,14].

The most common protocol of phage display technology can be summarized as: (1) preparing phage library e.g. random library and cDNA library; (2) immobilizing the target; (3) incubating the phage library with the immobilized target; (4) washing away the unbound phage with buffer; (5) eluting the bound phage with template or stronger buffer; (6) amplifying the eluted phage by infecting bacteria; (7) repeating steps (3)-(6) for more rounds; (8) amplifying the eluted phage and randomly picking up some clones for binding test and DNA sequencing. The foreign peptides displayed on the surface of virion can then be derived from the DNA sequences.

In the foregoing description, the target refers to the substance used to screen the phage library, which is also known as bait or selector; the template refers the natural partner binding to the target. The whole process of affinity selection is usually called biopanning or just panning. The acquired peptides mimicking the binding site on the template and binding to the target are defined as mimotopes. The term mimotope was first introduced by Geysen *et al.* to describe peptides able to bind to the antigen combining site of an antibody but different from the epitope inducing the antibody [15].

In fact, it was hard to get mimotopes using Geysen’s method because all their peptides were synthesized chemically. This situation has changed since the coming of the phage display technology. Mimotopes can now be obtained in a relatively cheap, efficient and convenient way, *i.e.* screening phage-displayed random peptide libraries with a given target. However, not all eluted phage clones are target-specific, because the target itself is only one component of the screening system [16]. From time to time, clones binding to contaminants in the target sample or other components of the screening system such as the solid phase (e.g. plastic plates) and the capturing molecule (e.g. streptavidin and secondary antibody) rather than binding to the actual target, are recovered with those target-specific binders during panning. Peptides displayed on these phage clones are called target-unrelated peptides (TUPs), a term coined recently by Menendez and Scott [16]. Since all the TUPs mentioned above are favored during the affinity selection process itself, they are categorized as the selection-related TUPs. There is also another category of target-unrelated peptides, called the propagation-related TUPs, which are favored because of increased infectivity or productivity during phage propagations [17,18,19].

Mimotopes obtained from phage display experiments are very valuable. First, these peptides are candidates for new diagnostics, therapeutics and vaccines. Second, they can be used to predict the networks or sites of protein-protein interactions. Therefore, bioinformatics studies on integrating all available mimotope data and developing more powerful tools for mimotope analysis are of great importance. In this review, we will summarize the special databases, algorithms, programs, web servers and their applications in the phage display area, focusing on the tools for mimotope-based epitope mapping.

## 2. Databases

As a high-throughput technology, the amount of peptide sequence data derived from phage display has been accumulating rapidly. However, the data related to phage display experiments has been scattered in separate resources for a long period of time. For example, the structure data of mimotopes were store in the PDB database; the sequence data of phage vectors might be stored in the GenBank; the mimotope data were stored in various reference databases with the published papers. Special databases for mimotopes and other associate information are urgently needed since data integration plays a fundamental role in bioinformatics. Available databases for mimotope data are listed in Table 1.

**Table 1:** Databases for mimotope data.

Databases	Availabilities	Highlights and comments	Refs
ASPD	http://wwwmgs.bionet.nsc.ru/mgs/gnw/aspd	First mimotope database	[20]
RELIC Peptides	http://relic.bio.anl.gov/relicPeptides.aspx	Small molecules-oriented	[21]
MOTIF	Available after signing an agreement	Integrated into EMT tool	[22]
PEPBANK	http://pepbank.mgh.harvard.edu	Not only mimotopes	[23]
MimoDB	http://immunet.cn/mimodb	Newest and largest	[24]

ASPD is short for Artificially Selected Proteins/Peptides Database. It is the first special database for mimotopes [20]. This database became available at the beginning of the 21^st^ century, collecting panning results from random libraries, mutant libraries and cDNA libraries. At present, the ASPD database contains data on 195 selection experiments, which were described in 112 original papers. For each experiment, the following information is given: target, template, links to the external databases such as SWISS-PROT and PDB, aligned sequences of peptides retrieved through *in vitro* evolution and relevant native or constructed sequences, rounds of selection, occurrences of clones with each sequence. For each paper, a full reference, a link to the MEDLINE database and the name of the corresponding author with his email address are recorded. All curated data are stored in two flat files. One is for panning experiment; another is for literature. ASPD has a user-friendly interface and can be searched by means of the SRS system. There is a BLAST search tool against the ASPD database for looking directly for homologous sequences. Regretfully, the ASPD database has not been updated for years.

The RELIC Peptides is a relational database created with ORACLE 9i and can be accessed through a web interface coded with ASP. It currently houses over 5,000 peptide sequences that have been selected with small molecule metabolites such as ATP, GTP and glucose and drugs such as Taxol and Taxotere, as well as random clones from parent libraries. As part of the RELIC suite, this database is indispensible because many programs in RELIC are dependent on the data of RELIC Peptides [21].

The MOTIF database contains 1,502 peptides obtained from the public domain and by competitive screening of phage display libraries using antisera raised against allergens and industrial enzymes [22]. It also contains 1,013 binding motifs derived from the sequence alignments of these mimotopes. This database was integrated into an epitope mapping tool (EMT), which can search motifs in the database against a given antigen structure. Matches indicate all possible epitopic regions on the antigen.

The PEPBANK database also includes some peptide data from phage display technology [23]. At the time of writing, it contains a total of 21,691 individual peptide entries. The major source of peptide sequence data comes from text mining of MEDLINE abstracts with a program coded with Perl. Another component of the PEPBANK database is the peptide sequence data from ASPD and UniProt. An additional, smaller part of the database is manually curated from sets of full text articles and text mining results.

The MimoDB database is the newest database for mimotopes, which is developed by our team very recently [24]. This database is scheduled to be revised and updated quarterly, collecting mimotopes from random libraries. Peptides with sequences longer than 40 residues or selected from phage display cDNA libraries (e.g. antibody phage display libraries) are not included. In the current release, it has 10,716 peptides manually curated from 571 publications, which were grouped into 1,229 sets. These peptides are selected with 775 different targets. The type of targets is quite diverse, varying from small compounds to nucleic acids, proteins, cells, tissues, organs and even entire organisms. Nonetheless, proteins including antibodies and receptors from human and mouse are the most used targets. At present, there are 257 known templates in MimoDB. For most of the peptides, their templates are not determined. The database also stores 53 solved structures for target-template complex, which are related to 63 mimotope sets. There are five solved structures for target-mimotope complex, which are related to four mimotope sets. For structures of target-template or target-mimotope complex, contact residues making the interface can be viewed interactively with JmolApplet in MimoDB. The MySQL relational database management system is used to store and manage all the data described above. The MimoDB database can be browsed and searched through a user-friendly web interface coded with PHP.

All the databases described here are important resources for the phage display community. With the large amount of sequences in these databases, it is feasible to find out new target-unrelated peptides. For example, in our preliminary analysis, among the 10,716 peptides in MimoDB, 9,802 peptides appear only once; 378 peptides appear 914 times [24]. SVSVGMKPSPRP, HAIYPRH and LPLTPLP are most frequent, which are seen in 22, 11 and 10 sets of mimotopes selected with different targets. SVSVGMKPSPRP and HAIYPRH have been proved to be propagation-related TUPs [17,18]. Experimental biologists can search the MimoDB database to verify if their results have appeared in the database. If so, the match might mean that different research groups have isolated the same peptide with different targets. In this situation, the peptide may not be a true target binder. It is also convenient for computational biologists to derive benchmarks and customized data sets from these databases, which are useful for new algorithm development and tool evaluation.

## 3. Algorithms, Programs and Their Applications

Mimotopes are considered to be similar with the corresponding template in some way since they bind the target competitively. It is only natural that the first thought would be their sequence similarity. It is true in some cases. Mimotope sequence might be very similar or even identical to one part of its template sequence, indicating the location of the protein-protein interaction site [2,25]. Eyes and tools are often used to align mimotope sequence to its template. Even if the template is not determined (e.g. panning against a small molecule drug with no priori knowledge about the corresponding drug target), a local alignment search against the non-redundant protein database with BLAST might help to find it out. However, most existing sequence alignment tools are developed and optimized for long protein sequences. They are not good at the analysis of short peptides deduced from phage display. Moreover, it is more often that the mimotope has little sequence similarity with its template, though they do have similar physicochemical properties and spatial organization locally. Therefore, many computational tools have been developed to interpret mimotope data reasonably onto the structure of its template. To improve the performance of these tools, algorithms and programs for excluding TUPs have also been developed. All these algorithms, programs and web servers are listed in Table 2. With these tools, phage display technology has shown its power in exploring the interactions between proteins, peptides and small molecule ligands.

**Table 2:** Computational tools for mimotope analysis.

Tool Names*	Availabilities	Highlights and comments	Refs
PEPTIDE	Not stated	Relies on other commercial tools	[26]
Ganglberger	Not stated	Coarse-grained 3-dimensional search	[27]
FINDMAP	Not stated	Independent of template structure	[28]
SiteLight	Not stated	First patch-based method	[29]
Mapitope	http://pepitope.tau.ac.il	First pairs-based method	[30,31]
RELIC	http://relic.bio.anl.gov	A suite of 14 computational tools	[21]
3DEX	http://www.schreiber-abc.com/3dex	First downloadable stand-alone tool	[32]
MIMOX	http://immunet.cn/mimox	First freely-accessible web server	[33]
MIMOP	Available upon request	Includes MimAlign and MimCons	[34]
EPIMAP	Not stated	Improved version of FINDMAP	[35]
PepSurf	http://pepitope.tau.ac.il	First graph-based method	[36]
Pepitope	http://pepitope.tau.ac.il	Server for Mapitope and PepSurf	[37]
Perschinka	Available upon request	Only tool coded with MATLAB	[38]
Pep-3D-Search	http://kyc.nenu.edu.cn/Pep3DSearch	Ant Colony Optimization algorithm	[39]
Denisova	Not stated	Based on the idea of Mapitope	[40,41,42]
EpiSearch	http://curie.utmb.edu/episearch.html	Impressive performance	[43]
SAROTUP	http://immunet.cn/sarotup	Detects target-unrelated peptides	[44]
MimoPro	http://59.73.198.183:8080/MimoPro	Fluctuating distance threshold	

* The name of the first author is used if the name of the computational tool is not available.

### 3.1. Tools for Exploring Protein-Protein Interactions

Generally, all methods described in this section can be divided into two categories, *i.e.* the sequence-sequence alignment category and the sequence-structure alignment category. FINDMAP, EPIMAP and the MimAlign method of the MIMOP belong to the first category. All methods left can be included in the sequence-structure alignment category, which requires mimotopes and the structure of template as input. In this category, there are motif-based methods (such as 3DEX, PEPTIDE, MIMOX, MIMOP and *etc.*), pairs-based methods (such as Mapitope and Denisova method), patch-based methods (such as SiteLight and EpiSearch), graph-based methods (such Pep-3D-Search and Pepsurf) and all kinds of hybrid methods (such as MimoPro). Though different from each other, most mentioned methods mainly address on deciphering the protein-protein interactions, especially epitope mapping.

Although specific peptides can be selected from the phage-displayed random peptide libraries with various targets ranging from small compounds to whole organisms, the most frequently used targets are proteins, especially antibodies. Actually, phage display technology was used for epitope mapping from its infancy [2]. Powered by special computational tools developed for phage display technology, not only linear epitope but also conformational epitope formed by discontinuous residues brought into spatial proximity by protein folding can be mapped reasonably.

The Tramontano lab from Italy might be the first team that tried to map discontinuous epitope computationally based on mimotopes and the antigen structure [26]. Their method includes four steps. First, the solvent accessible area of each residue of the antigen is calculated with the program *What if* and then surface residues were determined. Second, a program called PEPTIDE, which they developed, is used to create a file in PIR format containing all the peptide sequences of a given length that can mimic a preselected number of side chains exposed on the surface of antigen structure. Third, the commercial package such as GCG is used to search the PIR file with the consensus sequence obtained from a set of mimotopes. Last, energy minimizations and molecular dynamic simulations are performed with the commercial software packages such as Insight and Discover. As the commercial programs or the 3^rd^ party software were expensive and not integrated well enough, this method were not widely used by the phage display community.

In 2000, the Jensen-Jarolim group from Austria proposed a 3-dimensional epitope search method and applied it to localize the major IgE epitope on Bet v 1, the major birch pollen allergen [27]. In brief, the 3-dimensional coarse-grained epitope search was based on the X-ray structure of Bet v 1. Each amino acid in the structure was localized using the coordinates of its Cβ atom (for glycine, the Cα atom). Based on this Cβ grid, neighboring amino acids were found in a distance smaller than 6.1 Å. Fitting of only two amino acids consecutively induced a broader attempt, which allowed gaps. The model was further simplified by classifying all amino acids into four groups: polar, lipophilic, acidic and basic amino acids. All hits were statistically evaluated. Using this method internally, they found two regions on Bet v 1 surface significantly similar with mimotope, though aligning the mimotope with Bet v 1 sequence using the GCG package failed to find any similarities.

In 2003, Mumey *et al.* described the program FINDMAP [28]. This program aligns the mimotope sequence to its template sequence, allowing any permutations and local rearrangements (e.g. inversion) of the mimotope sequence. It is therefore different from traditional sequence alignment and has proved to be NP-complete. A branch-and-bound algorithm was used to solve this alignment problem in practice and implemented with C++ originally. FINDMAP is unique because it is only based on sequences. The sequences of mimotope and its template are enough; the structure of template is not needed. With this advantage, it has been widely used to explore the epitope and topology of membrane proteins, which lack solved structures due to difficulties in crystallization [45,46,47,48,49]. Recently, an improved version with the name EPIMAP has been proposed and coded with Java [35]. While FINDMAP can deal with only one mimotope or a consensus sequence, EPIMAP is capable of: (1) aligning each mimotope to the template and producing a set of the top-scoring alignments; (2) selecting the most mutually compatible alignments and filtering out spurious alignments with the program EPIFILTER.

The program SiteLight developed by Halperin *et al.* might be the first patch-based method [29]. Briefly, the template surface is divided into overlapping patches based on geodesic distances between two Cα atoms of amino acids; each mimotope is compared with each patch and a bipartite graph is created for each potential match scored by a similarity matrix; the best alignment of a mimotope and a patch represented in a bipartite graph is found by the maximal bipartite matching algorithm.

The Mapitope algorithm was originally proposed by Enshell-Seijffers *et al.* [30]. The core idea of Mapitope is that: (1) the simplest meaningful fragment of an epitope is an amino acid pair; (2) amino acid pairs on the template surface can be simulated by amino acid pairs in the mimotope sequence. Two amino acids on the template surface can be considered as a pair when the distance between their Cα atoms is less than a threshold, for example 8 Å. To predict the epitope with a set of mimotopes, at first, each peptide is converted to overlapping sequence pairs. All amino acid pairs derived from the set of mimotopes are then pooled, and the frequency of each type is calculated and it is determined whether its representation in the pool is higher than the random expectation. Once the most significant amino acid pairs of the pool are identified, the algorithm seeks the match pairs on the template surface and attempts to link them into clusters. The Mapitope program was implemented with C++ and has been applied in predicting epitope recognized by monoclonal antibodies against viruses [30,50,51].

As the above method typically predicts only a fraction of the epitope, Denisova *et al.* added a filling step to improve the performance of Mapitope [40]. In the filling step, an additional set of amino acid pairs (separated by distance no longer than 8 Å) for every predicted cluster based on the mimotope sequences, is created. This new dataset is then compared to the total amino acid pair composition of the mimotopes. New pairs that were present in the original dataset but were not selected initially because they did not meet the statistical threshold are indentified. The cluster analysis is then repeated using the new pairs and the largest clusters are predicted to be the epitope. The improved method was applied to the prediction of epitopes for five monoclonal antibodies against the West Nile virus E protein. In the case of E16 monoclonal antibody, only three contact residues were uncovered by the original algorithm, while additional nine contact residues were found by the improved algorithm. 

In our opinion, there is quite a lot information loss while converting mimotopes to overlapping sequence pairs linked with a peptidyl bond [52]. Other pairs, perhaps all pairs, should be considered. According to our observation, other conformations such as helix, turn, hairpin and even globular shape can be seen in the structures of free and bound mimotopes besides the extended conformation. This means that two residues far away in a mimotope sequence may fold together to form a space pair. Furthermore, based on our computations on 640 representative structures in the PDB database, the Cα distances between two residues separated by one amino acid in all types of conformations are below 8Å. This indicates that at least pairs separated by one residue in the mimotope sequence are space pairs and should not be ignored [52].

The same fact might have also been noticed by other groups. In a recent method based on pattern recognition theory, all possible space pairs (for example, pairs separated by one residue, two residues, three residues, and so on) in mimotope sequences are taken into account [41,42]. This new method can be regarded as a derivative of Mapitope. However, it is specially designed for elucidating epitope specificity within antiserum. The method consists of two phases: learning and identification. During the learning phase, a large set of mimotopes is collected through panning against specific monoclonal antibodies. The mimotopes are analyzed to identify epitope specific pairs. During the identification phase, mimotopes selected using patient antiserum are interrogated for the presence of the epitope specific pairs.

In 2005, the tool 3DEX (short for 3D-Epitope-Explorer) was developed by Schreiber *et al.* [32]. To localize a mimotope on the structure of its template, 3DEX maps each amino acid in the mimotope into a table, containing all the same amino acids on the template surface. The residues in each table are then connected one by one if their Cα or Cβ distances below the predefined threshold. Gaps are allowed in the connecting process. 

In 2006, a PHP program named MIMOP was developed by Moreau *et al.* [34]. It includes two methods. MimAlign, the first method, combines results from four multiple sequence alignments of the template and its mimotopes. For each position, a frequency and a score are calculated. Convergent positions are then selected and clustered based on their topology. The clusters obtained are considered as potential epitopic regions, then scored and ranked. The second method named MimCons, evaluates the similarity of the mimotopes and clusters them accordingly. Consensus patterns are identified from mimotope sequences of each cluster. The template surface is scanned to look for all possible exposed consensus patterns. The two methods can be run independently or their results combined.

MIMOX might be the first freely accessible web tool for mimotope-based epitope mapping [33]. It was coded with Perl using modules from the Bioperl project. MIMOX has two sections. In the first section, it provides a simple interface for ClustalW to align a set of mimotopes and a consensus sequence is derived from the alignments. In the second section, MIMOX can map a single mimotope or a consensus sequence, or part of them, onto the corresponding antigen structure and search for all of the clusters of residues that could represent the native epitope. NACCESS is used to evaluate the surface accessibility of the candidate clusters; and Jmol is embedded to view them interactively in their 3D context. MIMOX is an interactive rather than an automatic tool at present. The default parameters of the program are optimized to decrease the load of the server. Thus, the users often need to adjust the parameters many times to get a reasonable result. MIMOX has been applied by immunologists to characterizing both monoclonal antibody and antiserum [53,54].

In 2007, Perschinka *et al.* proposed a structural alignment method to identify conformational epitopes on heat shock protein 60 associated with atherosclerosis [38]. In their method, each mimotope is divided into a set of overlapping 5-mer peptides. These peptides are then superimposed onto the template surface according to Cβ atoms. An alignment score is calculated for every superimposition based on similarity of the superimposed amino acids and the distance between the superimposed Cβ atoms. For each 5-mer peptide, the calculated structural alignment score of each surface exposed amino acid is plotted. A control plot can be done with a random peptide. Peaks in the plot for mimotope with high scores can easily be observed and taken as the positive hits. The program was written in MATLAB 6.0 and the source code is available on request from the authors.

Mayrose *et al.* described a graph-based tool PepSurf for mapping a set of mimotopes onto the solved structure of the template [36]. In Pepsurf, the problem is converted into the task of aligning a set of query peptides to a graph representing the template surface. The best match of each mimotope is found by aligning it against virtually all possible paths in the graph. A clustering step then combines the most significant matches and a predicted epitope is inferred. The program was written in C++ and can be used directly through the Pepitope web server [37]. The Mapitope and a combination of PepSurf and Mapitope algorithm are also implemented in the Pepitope. The PepSurf algorithm and the Pepitope web server have been widely used in predicting epitopes on toxins, allergens and receptors recognized by monoclonal or polyclonal antibodies [55,56,57,58].

In 2008, Huang *et al.* proposed another graph-based tool Pep-3D-Search [39]. In this method, a surface graph of all exposed residues on the template is created at first. Then, the algorithm can be employed in two modes. The first mode is the mimotope mode, which searched for matching paths on the template surface with each query mimotope by the Ant Colony Optimization (ACO) algorithm. All paths were scored to the corresponding mimotope according to an amino-acid substitution matrix. Putative candidate epitopes were then picked out by the P-value calculation algorithm and the Depth-First Search algorithm. The second mode is the motif mode, which directly mapped the motif onto the template surface using the ACO algorithm and took the top-scoring paths as epitope candidates. All source code is in Visual Basic and can be downloaded freely.

In 2009, Negi *et al* proposed another patch-based method called EpiSearch [43]. With a set of mimotopes (up to 30 peptide sequences) and corresponding template structure as input, the algorithm first divides the surface of template into overlapping surface patches around each solvent accessible amino acid residue with a radius of 12 Å. Then it ranks all surface exposed patches according to the frequency distribution of similar residues in each mimotope and in each patch. EpiSearch is fully automated and has shown an impressive performance in the reported test cases.

The web server MimoPro has been available on line very recently. It is a mixture of the patch-based method and the graph-based method coded with Java. Firstly, the template surface is divided into overlapping surface patches centered at the Cα atom of each surface residue with a 15 Å radius. Then, the surface patches are converted into graphs by specifying two amino acids as neighbor amino acids using a fluctuating distance threshold guided by the compactness factor. For each patch, a complete search method is conducted to find the best alignment for each mimotope sequence. Dynamic programming and branch-bound methods are adopted to avoid repeating search and narrow the search space. The floating distance threshold used in MimoPro is quite new as all tools mentioned previously use a fixed distance threshold. 

### 3.2. Tools for Exploring Small Molecule-Protein Interactions

Different from the tools described above, the RELIC server was particularly designed for the study of the interaction of small molecules with proteins [21]. By analyzing the sequence of a protein and the sequences of small molecule affinity-selected, phage-displayed peptides, RELIC can predict proteins or some residues on the protein that bind to drugs, drug candidates and small metabolites.

RELIC is not a single program but rather a suite of computational tools. It currently includes 14 programs: DNA2PRO, AAFREQ, POPDIV, AADIV, INFO, DIVAA, MOTIF1, MOTIF2, CLOSEcon, HETEROalign, DistSim, MATCH, FASTAcon and FASTAskan (see Table 3). The DNA2PRO is designed to get mimotope sequence from the Ph.D.-12 and Ph.D.-C7C phage libraries of New England Biolabs. Any other library of interest is supported if provided with the start and end DNA sequences of the vector. AAFREQ, POPDIV, AADIV, INFO and DIVAA are designed to analyze the statistical properties of a peptide population. These data are particularly valuable when calculated in conjunction with randomly chosen members of the unselected library, as some propagation-related TUPs can be identified and subtracted. MOTIF1 and MOTIF2 are designed to identify weak sequence motifs within short peptide sequence populations. CLOSEcon, HETEROalign and DistSim use PDB file of the template as the basis for analysis of protein-ligand interactions. If the structure of the template is not available, MATCH, FASTAcon and FASTAskan can be used to do optimal sequence alignments between mimotopes and its template sequence. All programs in the RELIC suite were developed in FORTRAN with a DOS based, command-line user interface. The DOS based applications were then converted into web based applications by creating COM+ wrappers around the legacy code. As complicated software package online, RELIC, especially its tools for population analysis, motif identification and sequence alignment are extensively used by the phage display community [59,60,61,62,63,64,65,66,67,68,69,70,71,72,73]. 

**Table 3:** Computational tools in the RELIC suite.

Tool Names	Availabilities	Highlights and comments
DNA2PRO	https://relic.bio.anl.gov/dna2pro.aspx	Mimotope sequence from DNA sequence
AAFREQ	https://relic.bio.anl.gov/aafreqs.aspx	Frequency of each residue at each position
POPDIV	https://relic.bio.anl.gov/popdiv.aspx	Population diversity of mimotopes
AADIV	https://relic.bio.anl.gov/aafreqs3.aspx	Amino acid frequency and diversity
INFO	https://relic.bio.anl.gov/info.aspx	Likelihood of random occurrence
DIVAA	https://relic.bio.anl.gov/divaa.aspx	Sequence diversities and relationships
MOTIF1	https://relic.bio.anl.gov/motif1.aspx	Identifies continuous short motifs
MOTIF2	https://relic.bio.anl.gov/motif2.aspx	Identifies discontinuous short motifs
CLOSEcon	https://relic.bio.anl.gov/closecon.aspx	Finds contact residues for a small ligand
HETEROalign	https://relic.bio.anl.gov/hetero.aspx	Aligns a mimotope to a PDB file
DistSim	https://relic.bio.anl.gov/DistSim.aspx	Computes distance and similarity
MATCH	https://relic.bio.anl.gov/match.aspx	Aligns mimotopes to template sequence
FASTAcon	https://relic.bio.anl.gov/fastaconsen.aspx	Finds short consensus sequences
FASTAskan	https://relic.bio.anl.gov/fastaskan.aspx	Possible templates for a set of mimotopes

### 3.3. Tools for Excluding Target-Unrelated Peptides

As discussed previously, the results from phage display technology are noisy. Besides mimotopes, target-unrelated peptides often creep into and even dominate the biopanning results [16,17,18,19]. Although strict control and subtractive experiment might help to decrease TUPs, either selection-related TUPs or propagation-related TUPs can not be eradicated. Undoubtedly, taking TUPs as mimotopes would make the experimental and computational conclusions misleading. To improve the accuracy of programs for mimotope-based analysis, procedures or special tools for excluding TUPs have been developed. 

When peptide population of both affinity-selected library and unselected library are analyzed by the program INFO in the RELIC suite, the propagation-related TUPs can be identified and theoretically subtracted from the affinity-selected library. The method is based on the theory of information by Shannon. This virtual subtraction process is used not only by INFO, but is also an option in the RELIC programs HETEROalign, MATCH, and FASTAskan to reduce the noise in affinity-selected peptide sequences and amplify the signal, *i.e.* mimotope [21].

Noise filtering procedure has also been implemented at the level of amino acid pairs. In the algorithm proposed by Denisova *et al.* recently, three groups of peptides are used [41,42]. Group A is a collection of mimotopes isolated by affinity selection using a series of specific monoclonal antibody. Group B is the set of peptides obtained by antiserum, which needs characterizing. Group C consists of 102 irrelevant peptides to be used as a negative control. At the learning phase, the entire collection of Group A peptides is used to filter amino acid pairs that are common to more than one antibody.

We have developed a free web tool called SAROTUP, which can be used to scan, exclude and report possible target-unrelated peptides from mimotopes [44]. At present, a set of 23 TUP motifs collected from literature are compiled in the program. Among them, one motif indicates propagation-related TUPs; 22 motifs indicate selection-related TUPs, including 12 motifs specific for the capturing agents, five motifs specific for the constant region of antibody, three motifs specific for the screening solid phase and two motifs specific for the contaminants in the target sample. These motifs are converted to regular expressions and then used to check input peptide sequence one by one. However, there are a lot of target-unrelated peptides bearing no known motifs [16]. As these TUPs are not embedded in SAROTUP at present, it is possible that a true TUP cannot be detected by SAROTUP. One way to reduce such false negatives is to search the MimoDB database. As stated previously, analysis on peptides in the MimoDB database can also revealed new TUPs.

## 4. Problems and Prospects

The existing bioinformatics resources and tools reviewed in this paper have benefited the phage display community. However, there are still a lot of problems need to be solved. For example, known TUPs, especially propagation-related TUPs are very limited. Is there any pattern exists in propagation-related TUPs? Most tools ignore TUPs and only a few tools have integrated a filtering procedure. No tools can map epitope formed by two or more chains. The performances of available tools are far from satisfying, and *etc*. We believe all the mentioned problems will be solved in the coming years. With the advances of bioinformatics tools, the powerful phage display technology will become even more powerful. We can expect that some tools can also be used to other similar surface display technology such as ribosome display, yeast display and bacterial display, producing broader influence. 
